# Root hemiparasitic plants are associated with more even communities across North America

**DOI:** 10.1002/ecy.3837

**Published:** 2022-09-30

**Authors:** Jasna Hodžić, Ian Pearse, Evelyn M. Beaury, Jeffrey D. Corbin, Jonathan D. Bakker

**Affiliations:** ^1^ School of Environmental and Forest Sciences University of Washington Seattle Washington USA; ^2^ U.S. Geological Survey Fort Collins Science Center Fort Collins Colorado USA; ^3^ Organismic and Evolutionary Biology Graduate Program University of Massachusetts Amherst Amherst Massachusetts USA; ^4^ Department of Biological Sciences Union College Schenectady New York USA

**Keywords:** *Castilleja*, diversity, hemiparasite, parasitic plants, parasitism, species coexistence, species evenness, species richness

## Abstract

Root hemiparasitic plants both compete with and extract resources from host plants. By reducing the abundance of dominant plants and releasing subordinates from competitive exclusion, they can have an outsized impact on plant communities. Most research on the ecological role of hemiparasites is manipulative and focuses on a small number of hemiparasitic taxa. Here, we ask whether patterns in natural plant communities match the expectation that hemiparasites affect the structure of plant communities. Our data were collected on 129 national park units spanning the continental United States. The most common hemiparasite genera were *Pedicularis*, *Castilleja*, *Krameria*, and *Comandra*. We used null models and linear mixed models to determine whether hemiparasites were associated with changes in community richness and evenness. Hemiparasite presence did not affect community metrics. Hemiparasite abundance was positively associated with increasing evenness of herbaceous species, but not with species richness. The associations that we observed on a continental scale are consistent with evidence that the impacts of root hemiparasitic plants on evenness can be substantial and abundance dependent but that effects on richness are less pronounced. Hemiparasites mediate competitive exclusion in communities to facilitate species coexistence and merit consideration of inclusion in ecological theories of coexistence.

## INTRODUCTION

Understanding the mechanisms that maintain and promote species coexistence and community diversity has long held the interest of ecological researchers (Chesson, 2000; Comita et al., 2014). Some groups of organisms affect species diversity and ecosystem functioning through their unique morphological and physiological attributes. These functional plant types can have disproportionate impacts on their communities, affecting niche partitioning and species coexistence (Violle et al., 2007). One well‐known example is the case of nitrogen‐fixing plants, which facilitate nitrogen cycling and stimulate the growth and productivity of communities as a whole (Craine et al., 2002; Temperton, et al., 2007).

Parasitic plants are another important functional group of plants known to affect community structure (Yoshida, et al., 2016). These plants attach to host plants via specialized structures known as haustoria. Holoparasites, which cannot photosynthesize and rely entirely on host plants, have been compared to specialist herbivores because they exhibit a narrow host range (Pennings & Callaway, 2002). The Janzen–Connell hypothesis, which provides a mechanism for species coexistence by which specialized natural enemies regulate plant species in a density‐dependent way (Connell 1971; Janzen, 1970), has been extended to some holoparasites (Grewell, 2008). In contrast, root hemiparasites, parasitic plants that have retained the ability to photosynthesize, have largely been ignored in ecological theory, even though they are globally ubiquitous (Těšitel, 2016). Unlike holoparasites, hemiparasites are generalists and attach to and parasitize a wide variety of hosts simultaneously (Marvier, 1998; Matthies, 2017). However, host defense and tolerance can vary dramatically, as can intra‐ and interspecific hemiparasite effects and dependence upon hosts (Hejduk, et al., 2020; Li, et al., 2012; Press & Seel, 1996). Furthermore, hemiparasites have a vestigial and shallow root system, and a significant factor determining whether a host is parasitized is the host's belowground dominance and proximity to the hemiparasite. Consequently, in natural communities, suitable host species that are also dominants are disproportionately parasitized by hemiparasites (Press & Phoenix, 2005).

The potential impacts of hemiparasites on communities aligns with a growing body of literature demonstrating that organisms with a parasitic lifestyle act as facilitators to alter patterns of co‐occurrence and trophic complexity (Hatcher et al., 2012). For example, because many parasites can serve as both predators and prey, they play an outsized role in food webs relative to their biomass (Haan et al., 2018; Lafferty et al., 2006). Parasites can also influence the outcome of trophic interactions at a community scale (Hudson et al., 2006). For example, by reducing the fitness of their hosts, parasites can make them more susceptible to predation, disease, or competition. Finally, parasites can positively contribute to biodiversity by allowing competitively inferior species to persist (Price & Westboy, 1986).

Variable performance with different hosts, together with density‐dependent encounters with hosts, positions hemiparasites to alter community structure (Phoenix & Press, 2005; Těšitel, 2016). Hemiparasite addition and removal experiments have shown that hemiparasites can increase the evenness and richness of their communities (Borowicz & Armstrong, 2012; Grewell, 2008; Těšitel et al. 2017). By reducing host growth, hemiparasites can alter competitive dynamics between host and nonhost species, potentially increasing the growth and abundance of subordinate species. They have even been shown to impact trophic levels beyond plants (Haan et al., 2018; Hartley et al., 2015) and to alter nutrient cycling (Fisher et al., 2013; Spasojevic & Suding, 2011; Quested, et al., 2003). Thus, because of their unique physiology, these plants can have a disproportionate effect on their ecosystem relative to their abundance and are considered keystone species and ecological engineers.

Community structure includes species richness and species evenness, which have been studied as separate response metrics. There is a growing appreciation that the effects of hemiparasites on evenness are likely linearly related to their abundance, whereas the positive effect of a hemiparasite on community richness peaks at intermediate abundances of hemiparasites (Fibich et al., 2017; Heer et al., 2018; Hejduk et al., 2020). Thus, we expect sites where hemiparasites are present and abundant to have more even and species‐rich plant communities. Still, these differential patterns suggest that the mechanisms behind a hemiparasite's effect on evenness and richness differ and that our conclusions on how a hemiparasite affects its community depends on the response that is being considered. For example, a hemiparasite could increase richness by providing new niches for novel species to colonize or decrease richness by causing extirpation of present taxa. Because any change to richness requires either the gain or loss of a species, a hemiparasite's effects may be more mechanistically related to evenness.

Although there is strong experimental evidence in a few systems that hemiparasites affect plant community structure in a manner disproportionate to their abundance, it remains unclear whether those systems are representative of the full range of hemiparasites and plant communities in which they are found. Notably, much of our understanding of a hemiparasite's impact on its community has developed from experiments with annual hemiparasites in the genus *Rhinanthus*. In general, these studies point to a positive association of hemiparasites with evenness and richness (Ameloot, et al., 2005; Phoenix & Press, 2005), though exceptions are not uncommon (Ameloot, et al., 2006; Callaway & Pennings, 1998; Gibson & Watkinson, 1992). Furthermore, our current knowledge of the effect of hemiparasites is based primarily on work conducted in grasslands and prairies (Těšitel, et al., 2015). Because hemiparasites penetrate herbaceous host tissue more easily than woody tissue (Mudrák, et al., 2016), we expect their effects to be more pronounced in communities dominated by herbaceous rather than woody hosts. However, although hemiparasites generally prefer open and nutrient‐poor ecosystems (including alpine habitats, grasslands, or semiarid ecosystems), they are also found in habitats where many co‐occurring species (and, therefore, potential hosts) are woody (Nickrent, 2002; Těšitel, 2016). Despite this, their impacts in woody communities have not been studied.

Observational studies that look for the signatures of experimentally demonstrated ecological processes can help assess the scope of those processes (Adler et al., 2011; Beaury, et al., 2020; Sofaer, Jarnevich, & Pearse, 2018). In the case of hemiparasites, experiments form the clear expectation that hemiparasites can increase both the evenness and richness of plant communities (Těšitel et al., 2017). However, we must be cautious when connecting processes to ecological patterns. First, numerous aspects of the environment affect plant community richness and evenness, and those must be accounted for when looking for plant community associations with hemiparasites (Beaury et al., 2020). Second, most hemiparasitic plants are relatively uncommon in plant communities. Commonalities among species‐abundance relationships could result in spurious associations of an uncommon species with even or rich communities. Therefore, comparing hemiparasites with nonhemiparasitic plants of similar abundance may be important in corroborating or rejecting functional links between hemiparasites and plant community evenness and richness.

Here we analyze plant communities across North America, using plots from the U.S. National Park Service (NPS) that span a variety of ecoregions. After accounting for differences in plant communities based on ecoregions and management units, we ask (i) whether hemiparasite presence is correlated with increased evenness and richness of three groups of plants: all plants, herbaceous plants, and woody plants (hereafter we use the term “growth forms” to refer to these three groups), (ii) whether greater hemiparasite abundance is associated with increased evenness and richness in these plant communities, and (iii) whether associations of hemiparasite abundance and presence with plant community evenness and richness are greater than expected for similarly uncommon plant species. We demonstrate that high hemiparasite abundance is associated with more even herbaceous plant communities, but not more species‐rich plant communities, suggesting that some, but not all, experimentally demonstrated processes have left their fingerprints at a continental scale. We also demonstrate that hemiparasite presence is not associated with more species‐rich or even communities, suggesting that the purported relationships between hemiparasites and richness may be a statistical artifact, species‐specific, or the result of other mechanisms, like species pool effects.

## METHODS

### Plot survey data

Our data come from a large‐scale vegetation monitoring project designed to develop a uniform hierarchical vegetation classification standard for generating vegetation maps for lands managed by the NPS (Beaury et al., 2020; United States Geological Survey, 1994). Each plot included a list of all observed plant species, their percentage cover, and stratum class. Each plot was surveyed once, often between 1997 and 2012.

**FIGURE 1:**
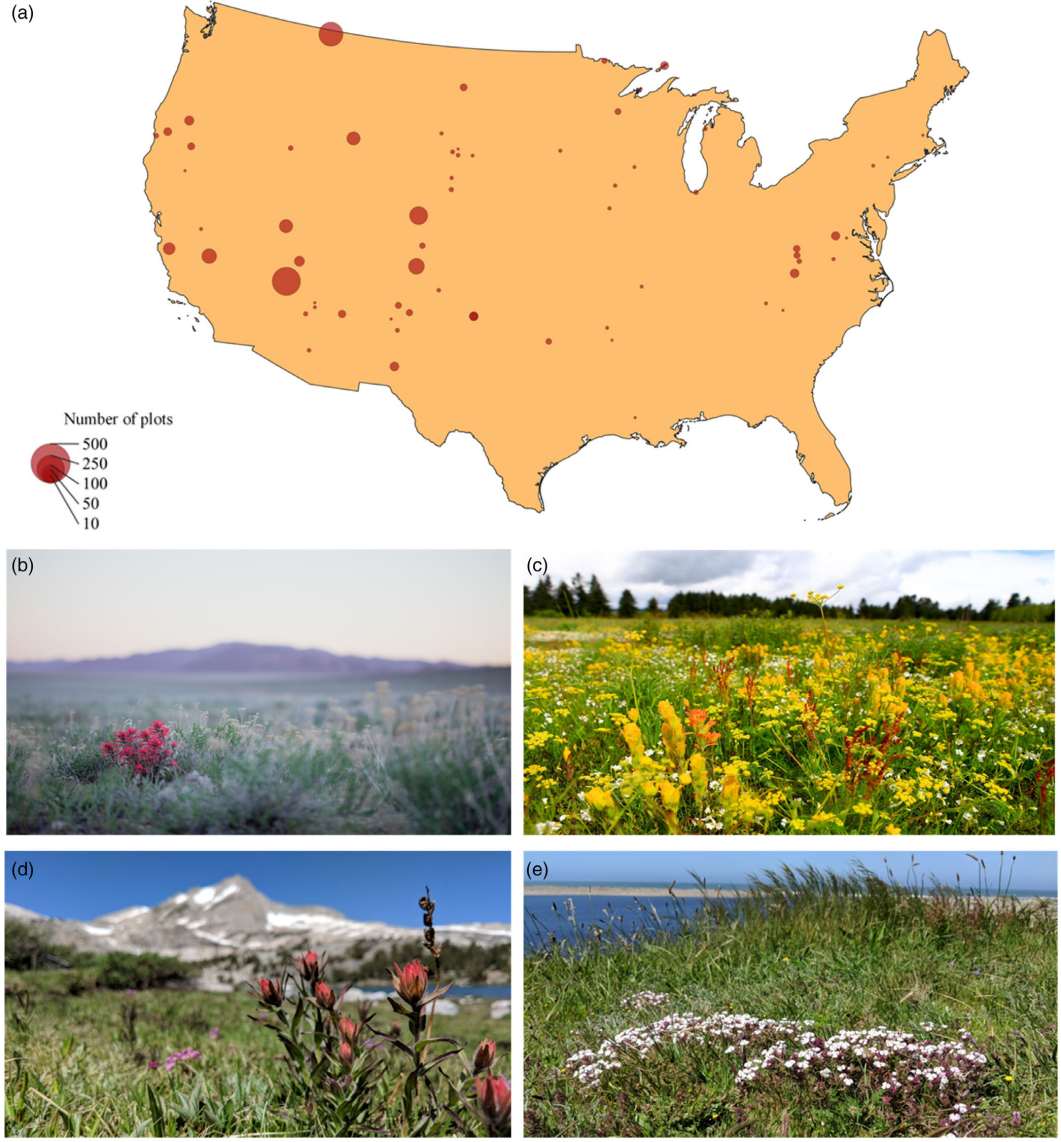
(a) Map of conterminous United States. Red circles indicate the location of 66 National Park Service management units that contain one or more hemiparasites, scaled by the number of total plots per management unit. A map showing all plots and management units is included in Appendix S1. (b) *Castilleja chromosa* in high sagebrush steppe of Nevada. (c) *Castilleja levisecta* and *Castilleja hispida* in Puget Sound prairies of Washington state. (d) *Castilleja miniata* outside Yosemite National Park. (e) *Triphysaria floribunda* in Point Reyes National Park. All images by Jasna Hodžić.

We assigned each plot (*n* = 25,151) to the ecoregion in which it was located, as defined by the Environmental Protection Agency (Omernik & Griffith, 2014). Ecoregions are identified by “analyzing the patterns and composition of biotic and abiotic phenomena that affect or reflect differences in ecosystem quality and integrity” (Omernik, 1987, 1995). We used the most detailed ecoregions (Level IV) to account for as much environmental variation as possible. Plots without recorded latitude and longitude (*n* = 4024) were excluded, leaving 21,127 plots across 220 Level IV ecoregions and 129 management units (Figure 1).

Plot area ranged from 1 to 5400 m^2^, depending upon the community type and park size. More than half of the plots were 400 m^2^; we restricted our analyses to these plots (*n* = 12,498 plots) to control for the relationship of area with species richness.

Our data set of 12,498 plots included 6987 plant species. We used the United States Department of Agriculture PLANTS Database (USDA, NRCS, 2022) to identify the growth habit of each plant species. We classified species as herbaceous or woody based on growth habit: Herbaceous included the growth habits of “forb/herb,” “graminoid” and “subshrub,” while woody included the growth habits of “tree” and “shrub.” All hemiparasites were classified as herbaceous. Taxa without a listed growth form were excluded from all analyses. We summed the covers of all taxa within each growth form; henceforth, we refer to this as abundance. Although abundance can be expressed in many ways, we assume here that increases in percentage cover are correlated with increases in belowground abundance. Finally, we calculated richness and the Shannon evenness index for each growth form and all plants in each plot. About an eighth of the plots (1608 of 12,498) contained at least one hemiparasite taxon. All plots that contained hemiparasites also contained nonhemiparasitic herbaceous plants (richness range of 1–79), and most (1551) contained woody plants (richness range of 1–28).

There were 12 hemiparasitic genera in our data set (out of ~22 native and nonnative genera in the United States)—*Aureolaria*, *Castilleja*, *Comandra*, *Cordylanthus*, *Dasistoma*, *Euphrasia*, *Geocaulon*, *Krameria*, *Orthocarpus*, *Triphysaria*, *Pedicularis* and *Seymeria*, but the most abundant genera are *Pedicularis* (32% of total hemiparasite abundance; family Orobanchaceae), *Castilleja* (25%; Orobanchaceae), *Krameria* (20%; Krameriaceae), and *Comandra* (13%, Santalaceae). The remaining genera each compose no more than 4% of total hemiparasite abundance. Overall, the data set included 78 unique hemiparasite taxa.

### Statistical analysis

We conducted all analyses in R version 4.0.2 (R Core Team, 2020). We considered hemiparasite presence by comparing plots with and without hemiparasites and considered hemiparasite abundance by focusing on the plots that contained hemiparasites. We began with a null model approach to compare the effects of hemiparasite presence and abundance with those of equally rare plants. If the null model analysis revealed trends, we analyzed hemiparasite‐specific models using linear mixed models. Finally, we divided the data set into subsets examine whether the 10 most abundant hemiparasites showed different associations than hemiparasites in abundance ranks 11 to 20 and to assess the genus‐specific effects of *Pedicularis* and *Castilleja*.

We began by considering whether hemiparasites might be associated with more even or rich communities solely because of their position in the rank‐abundance distribution of communities. Specifically, hemiparasites are relatively uncommon at small spatial scales, and it is possible that uncommon plants might be associated with even or species‐rich communities irrespective of their role in those communities (Jenkins, 2011; Weiher et al., 1998). We generated a rank‐abundance curve for all 6987 species in the data set. The ranks of the 78 hemiparasite taxa ranged from 795 to 6895, with most clustered toward the lower ranks and higher abundances (Appendix S1: Figure S1). We randomly designated 78 nonhemiparasitic taxa that also fell within this range as null model focal species.

For presence models, we paired plots globally such that each plot was paired with the closest plot within the same Level IV ecoregion. We discarded 3362 plots in which the closest neighbor was itself closer to a different plot and removed 996 plots in which the latitude and longitude of paired plots were identical. This resulted in 4070 plot pairs (8140 plots). The maximum distance was ~132 km (*n* = 2), and the median distance was <0.5 km. We drew 1000 random sets of plants from the global rank‐abundance curve of plant species inhabited by hemiparasites to create an empirical null distribution of model effect. The range of plots for each null plant species was 634 to 1674 plots. For both hemiparasite communities and null plant communities, we ran linear mixed models with the lme4 package (Bates, et al., 2015) relating each response variable (all evenness, all richness, herbaceous evenness, herbaceous richness, woody evenness, woody richness) to hemiparasite (or null hemiparasite) presence, with pair identity and sampling year specified as random effects. We log transformed the richness metrics for normality and transformed the evenness metrics with the logit function because evenness is bounded between 0 and 1 (Lesaffre, et al., 2007). We calculated statistical significance as the proportion of null models with a coefficient equal to or greater than that for hemiparasites. Our null model analysis did not indicate patterns were associated with hemiparasite presence (see *Results*), so we did not interpret the hemiparasite models in more detail.

For abundance models, we ran the same procedure to determine null species, but we did not pair plots together based on distance. We selected plots that contained at least one of the null model focal plants and calculated the six response variables for each plot (all evenness, all richness, herbaceous evenness, herbaceous richness, woody evenness, woody richness). We then ran linear mixed models with hemiparasite abundance as a fixed effect and sampling year and park identity and Level IV ecoregion as random effects. We applied the same transformations to the response variables as above and log transformed hemiparasite abundance for normality. We repeated both the random plot selection and designation of null model species 1000 times to create an empirical null distribution of model effects. We compared this empirical null distribution of model effects to the effects of hemiparasites to determine whether hemiparasite abundance had a greater association with plant community metrics than plants of comparable abundance. Our null model analysis indicated that some patterns were associated with hemiparasites (see *Results*), so we proceeded to assess the importance of hemiparasites by comparing the Akaike's information criterion (AIC) of the full model and the model without the hemiparasite abundance term.

To assess whether patterns associated with abundance were driven by the most abundant hemiparasites, we repeated the abundance analysis for the 10 most abundant hemiparasites (ranks 795–1691 in the data set) and for the hemiparasites in abundance ranks 11–20. Finally, to assess genus‐specific patterns for the most abundant genera, *Castilleja* and *Pedicularis*, we ran the same null analyses on data sets restricted by hemiparasite genus identity.

## RESULTS

### Hemiparasite presence

**FIGURE 2:**
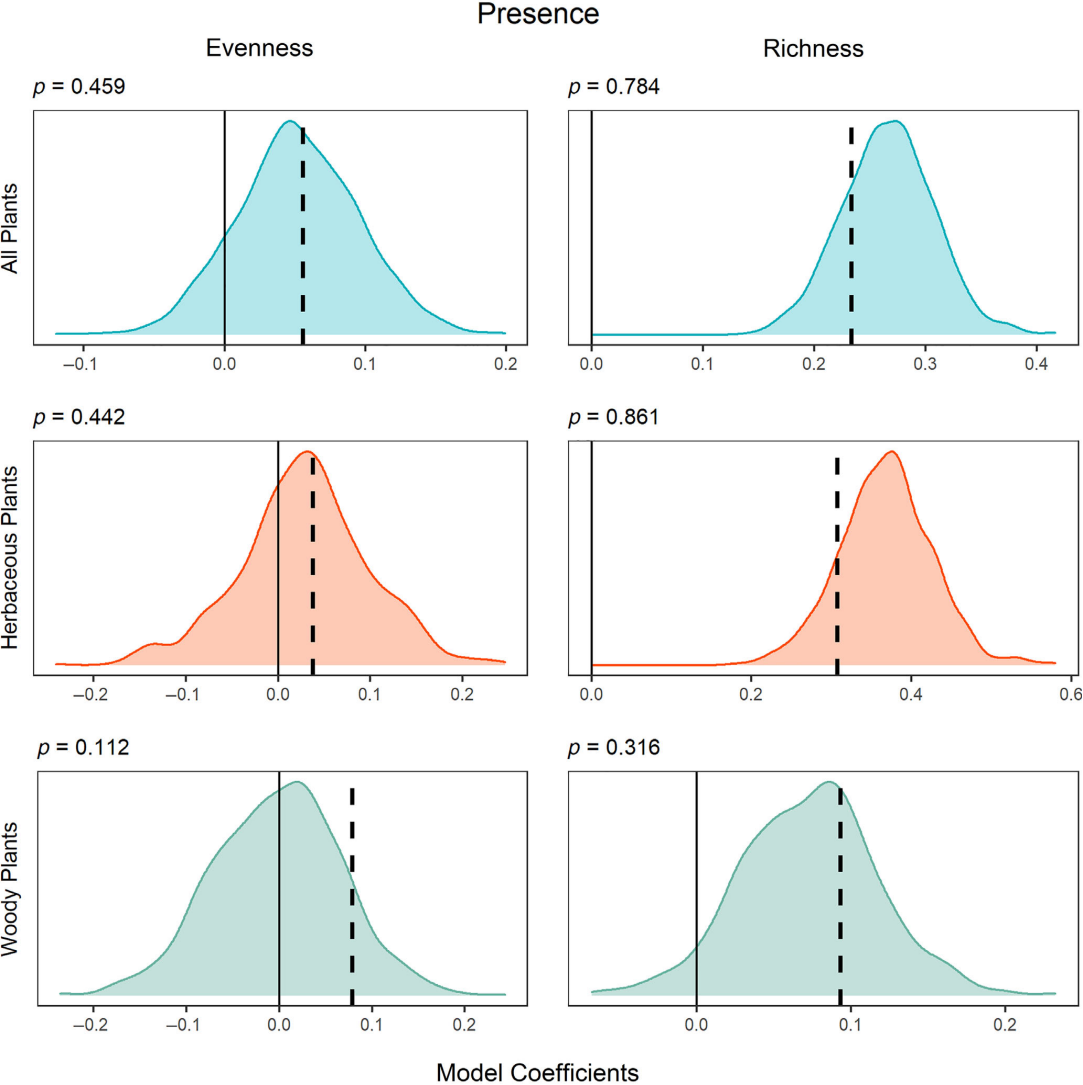
Density graph of model coefficients for 1000 null models created from plants in same rank abundance as hemiparasites for community evenness (left) and richness (right) for all plants (top row), herbaceous plants (middle row), and woody plants (bottom row). The *y*‐axis limits are identical across rows. The dashed line indicates the model coefficient for hemiparasites; positive coefficients indicate a positive relationship between hemiparasite presence and the response; negative coefficients indicate a negative relationship. Statistical significance was calculated as the proportion of null models with a coefficient equal to or greater than that for hemiparasites and is reported above each graph. The solid line at zero indicates no relationship between focal plant presence and the response.

The evenness and richness of all growth forms between paired plots were not related to hemiparasite presence any more than they were to the presence of comparable nonhemiparasites (Figure 2).

### Hemiparasite abundance

**FIGURE 3:**
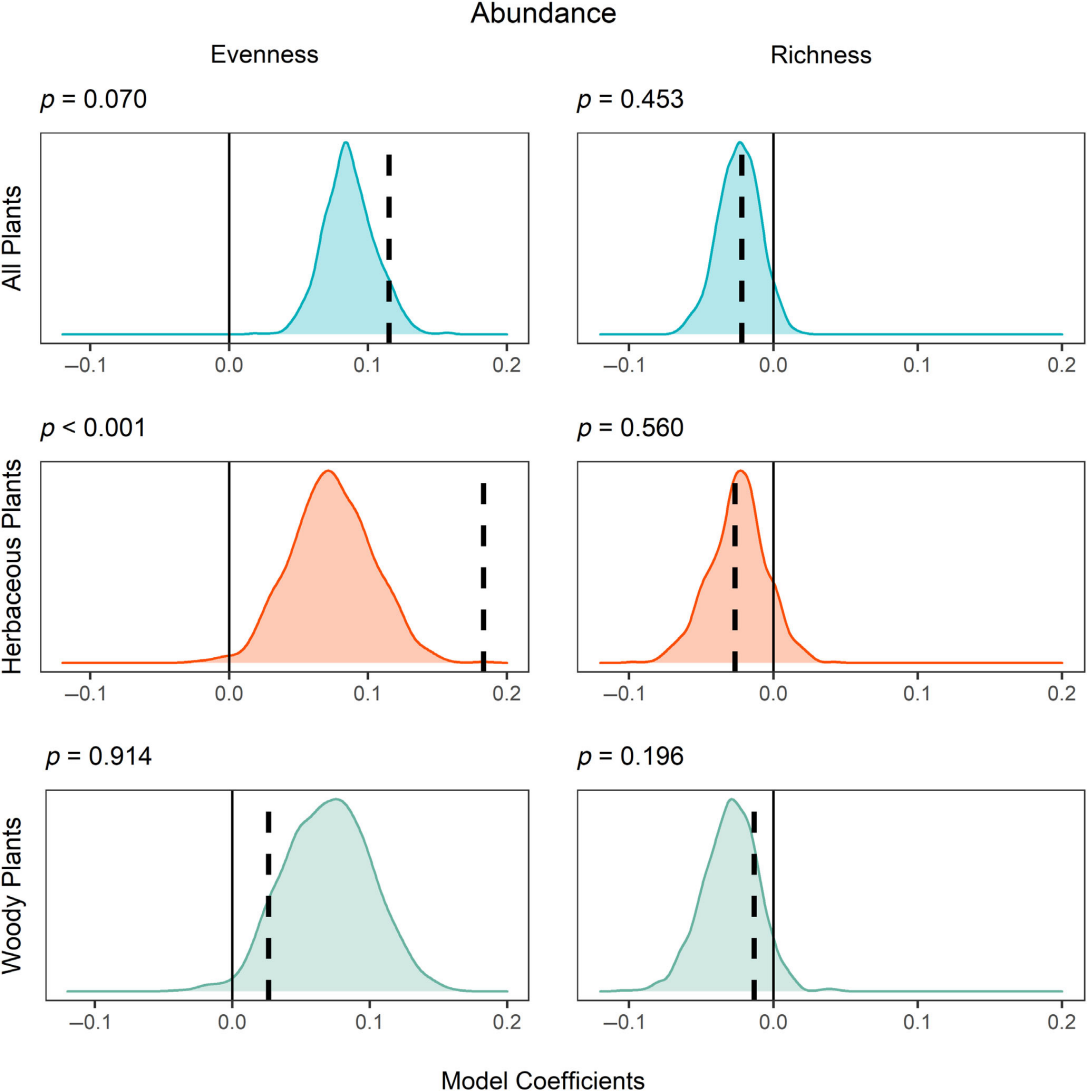
Density graph of model coefficients for 1000 abundance null models created from plants in the same rank abundance as hemiparasites for community evenness (left) and richness (right) for all plants (top row), herbaceous plants (middle row), and woody plants (bottom row). The *y*‐axis limits are identical across rows. The dashed line indicates the model coefficient for hemiparasites; positive coefficients indicate a positive relationship between abundance and the response; negative coefficients indicate a negative relationship. Statistical significance was calculated as the proportion of null models with a coefficient equal to or greater than that for hemiparasites and is reported above each graph. The solid line at zero indicates no relationship between focal plant abundance and the response.

The greater evenness of herbaceous plants was much more strongly associated with hemiparasite abundance than it was with the abundance of comparable nonhemiparasites (*p* < 0.001; Figure 3). Patterns were marginal for the evenness of all plants (*p* = 0.070) and highly nonsignificant for the evenness of woody plants. The richness of all growth forms was not related to hemiparasite abundance any more than it was to the abundance of comparable nonhemiparasites (Figure 3).

**FIGURE 4:**
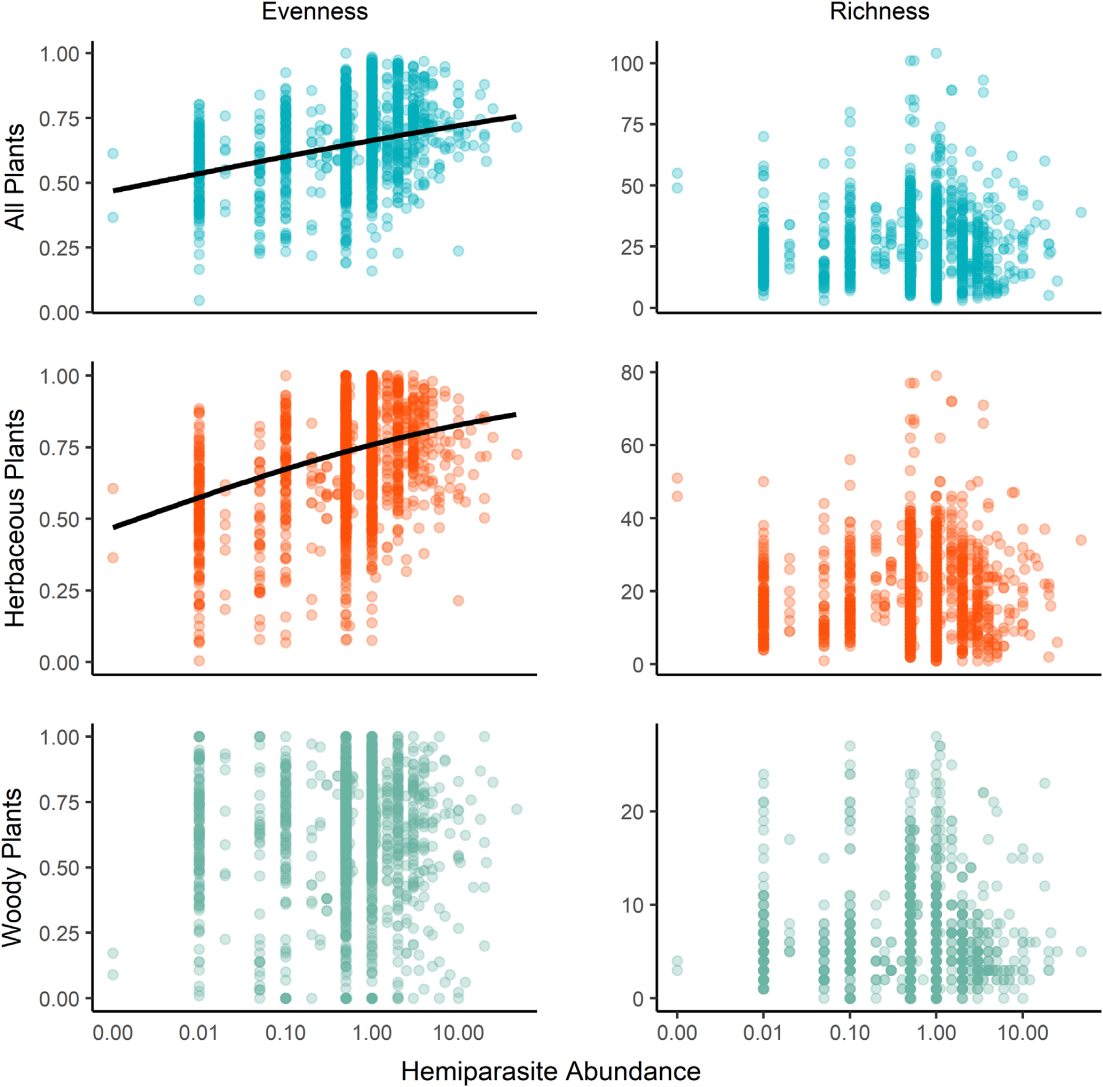
Relationship of hemiparasite abundance (total cover of hemiparasites) to community evenness (left) and richness (right) of all plants (top row), herbaceous plants (middle row), and woody plants (bottom row). The *x*‐axis was log‐transformed for visualization and in the analysis. Note that the scale of the *y*‐axis varies among graphs. Fit lines are only shown for significant trends. Model results are shown in Table 1.

Hemiparasite abundance was significantly correlated with increases in evenness for all plants and herbaceous plants (ΔAIC of 113 and 106, respectively), but not for woody plants. In contrast, hemiparasite abundance was not associated with a change in richness for any growth form (Table 1, Figure 4).

**TABLE 1:** Abundance (defined as sum cover) model results for hemiparasitic plants in national parks in the conterminous United States. Delta Akaike's information criterion (ΔAIC) is the difference between models with and without hemiparasite abundance as a predictor. ΔAIC values equal to or greater than two are significant and are bolded.

Predictor	Response	Growth form	*n*	Estimate	Marginal *R* ^2^	Conditional *R* ^2^	ΔAIC
Log hemiparasite abundance	Logit evenness	All plants	1608	0.117	0.060	0.55	113
		Herbaceous plants	1600	0.186	0.060	0.50	106
		Woody plants	1528	0.023	0.001	0.26	−6.72
	Log richness	All plants	1608	0.021	0.003	0.57	−2.90
		Herbaceous plants	1608	0.031	0.003	0.51	−3.26
		Woody plants	1608	0.018	0.001	0.64	−6.68

*Note*: Level IV ecoregion, year, and park identity were included as random effects. Sample sizes for evenness and richness are not always equal because plots with one taxon for a growth form have a defined richness but undefined evenness. Marginal *R* ^2^ provides the variance explained only by fixed effects; conditional *R* ^2^ provides the variance explained by the entire model. Response variables were transformed, as indicated, for analysis.

### Genus‐specific effects

The top 10 most abundant hemiparasites were significantly correlated with increases in evenness of herbaceous plants (*p* < 0.001) but not with any of the other responses (Appendix S1: Figure S3). The next most abundant hemiparasites (ranks 11–20) only showed a weak association with herbaceous evenness (*p* = 0.056; Appendix S1: Figure S4).

Species in the genus *Castilleja* were not associated with evenness but showed a positive association with increases in the richness of all plants (*p* = 0.029) and of herbaceous plants (*p* = 0.027; Appendix S1: Figure S5). Species in the genus *Pedicularis* were not correlated with any of the responses (Appendix S1: Figure S6).

## DISCUSSION

We have shown that some, but not all, of the experimentally demonstrated patterns related to the abundance of hemiparasites are detectable at a very large spatial scale. In particular, (1) hemiparasite abundance is more strongly associated with evenness than is the abundance of nonhemiparasites of similar rank, (2) evenness increases with hemiparasite abundance, and (3) hemiparasite presence is not more associated with species richness or species evenness plots than the presence of nonhemiparasite species of similar rank. Our results suggest that the correlation of hemiparasites with richness may be due more to a hemiparasite's distribution and habitat presence than to any stabilizing mechanisms associated with a parasitic lifestyle.

### North American hemiparasites can increase species evenness

Our null analysis demonstrated that any association between hemiparasites and more even herbaceous communities is density dependent. Additionally, the top 10 hemiparasites are more associated with even herbaceous communities (Appendix S1: Figure S3), following the pattern of all hemiparasites (Figure 3). However, the weaker effect for hemiparasites in ranks 11 to 20 (Appendix S1: Figure S4) further suggests that the impact of hemiparasites on evenness is abundance dependent.

Traditionally, the mechanisms of species coexistence have been organized into stabilizing forces, which increase niche differences between species, and equalizing forces, which reduce fitness differences between species (Chesson, 2000; Comita et al., 2014). Hemiparasites are thought to influence evenness and richness by disproportionately attaching to dominant hosts and reducing their growth to such an extent as to relax competitive exclusion of other subordinate species. This would reduce fitness differences between host and nonhost plants and exert an equalizing force on coexistence. This and other mechanisms by which a hemiparasite can act to influence evenness and impact species coexistence are promising avenues for future research. For example, the Janzen–Connell hypothesis provides an additional mechanism of coexistence by which specialized naturalist enemies can reduce the abundance and growth of a particular host plant in a density‐ or distance‐dependent way (Connell, 1971; Janzen, 1970). Although hemiparasites are considered to be generalists, they demonstrate host preference, selectively parasitizing only a subset of the hosts available to them and performing better with certain species (Gibson & Watkinson, 1992; Matthies, 1996; Shen et al., 2006). In this sense, the Janzen–Connell mechanism can also be extended to hemiparasites. Still, the community impact of a hemiparasite is predicated upon its ability to negatively impact its host. Several studies have shown that hemiparasites in *Castilleja* and *Pedicularis* can substantially damage their host (Kilgore, 2017; Matthies, 1997; Van Hoveln, et al., 2011), providing more support to the idea that these North American genera can affect community evenness. Our results for all hemiparasites also match those of the observational presence study of McKibben and Henning (2018), which showed an association of *Castilleja* with evenness, as well as the observational study of Fibich et al. (2017), which demonstrated a correlation between the presence of 16 hemiparasite species, two of which were in the genus *Pedicularis*, and more diverse plots. Furthermore, the abundance‐dependent effect of the hemiparasites supports the idea that a hemiparasite's effect is additive, that is, the greater the abundance of hemiparasites, the greater the effect on the community and, plausibly, the dominant host species. This result is also in line with Těšitel et al. (2016), who found that community evenness was linearly related to *Rhinanthus* abundance.

Mechanistically, the link between hemiparasite abundance and community evenness is due to either a reduction in host abundance due to parasitism or an increase in subordinate abundance as they are released from competitive exclusion. The relative importance of each of these mechanisms—that is, a decrease in dominant abundance versus an increase in subordinate abundance—could be the subject of future research. In addition, it is important to note that, for a root hemiparasite, belowground dominance is more important than aboveground dominance. These two are likely directly proportional in the open, nutrient‐poor ecosystems preferred by hemiparasites, like grasslands, though future research could clarify the extent to which above‐ and belowground abundance varies among species (Hiiesalu et al., 2012).

### Hemiparasites have unclear effects on richness

In contrast to evenness, our study showed no relationship with hemiparasite abundance or presence and community richness, except for an association between *Castilleja* abundance and richness (Appendix S1: Figure S5). These findings contradict most of the literature, including studies incorporating abundance‐dependent effects. For example, one study found that community richness was highest at intermediate densities of *Rhinanthus* and then declined as *Rhinanthus* density increased (Heer et al., 2018). However, we found that hemiparasite associations with richness were the same as those of other species that are similarly abundant in our data set, implying that apparent relationships with richness may be more a function of their distribution. It is important to note that a potential bias of the null models is that hemiparasites span a large range of rank abundances yet are clustered predominantly toward the lower ranks (Appendix S1: Figure S1). Thus, we may be artificially heightening their rarity by giving each rank equal weight.

Whereas a hemiparasite effect on evenness is predicated upon a stabilizing impact on competitive dynamics, to increase plant richness, a hemiparasite must facilitate the opportunity for colonization or increase the persistence of subordinate, competitively inferior species. In fact, we found a strong relationship between richness and the presence of nonhemiparasites of comparable rank abundance (Figure 3), suggesting that observed patterns in other studies, not just those focused on hemiparasites, may be statistical artifacts. Additionally, two biological mechanisms may explain a hemiparasite's association with richness (Fibich et al., 2017). First, hemiparasites are poor competitors for above and belowground resources. Hence, they likely perform best in species‐rich habitats that promote coexistence, rather than species‐poor habitats that may be stressed or provide limited resources. Second, hemiparasites also tend to occur in areas with large species pool effects, like grasslands. Any relationship with species richness, then, may be related to these habitat or species pool effects (Eriksson, 1993). Unfortunately, our data did not include detailed information on specific habitat type, although we used ecoregions to provide habitat information. However, our landscape‐level analysis provides evidence that the two aforementioned mechanisms are primarily responsible for a hemiparasite's association with richness.

### Effects are centered on the herbaceous components of communities

The insignificant associations between hemiparasites and woody plants make it unlikely that hemiparasites are having pronounced effects in woodland or forest ecosystems. Moreover, hemiparasites more successfully penetrate forb and graminoid hosts with fibrous, diffuse root systems (Mudrák et al., 2016). Thus, our results are in line with current understandings of hemiparasite plant biology.

### The role of life history

Unlike *Rhinanthus* and related genera, the dominant hemiparasitic genera in our study are mostly perennials: 94% of total hemiparasite abundance was provided by perennials. Furthermore, a hemiparasite's longevity may inform its resource acquisition strategies (Hodžić, 2021). Life‐history theory suggests that annuals should prioritize resource acquisition, whereas perennials benefit most from resource conservation (Friedman & Rubin, 2015). Thus, an annual hemiparasite may have a strong incentive to damage or even kill a host for the rapid acquisition of resources, whereas a perennial would benefit most by conserving host resources and inflicting less damage to hosts (Lepš & Těšitel, 2015).

Given life history's theorized role in mediating the host–hemiparasite relationship, as well as the obvious links between distribution and life history, investigating the link between a hemiparasite's longevity and its ultimate upstream effect on community productivity remains a promising area of future research. In this study, it was difficult to analyze the role of life history, for two reasons. First, there were simply not enough comparable plots with abundances of annual and perennial hemiparasites. Second, some plots contained both annuals and perennials, complicating comparisons. Finally, genera such as *Castilleja* contain both annual and perennial species, but these taxa were sometimes not identified to species, making it difficult to determine life history and to investigate single‐species trends. In our study, *Castilleja* abundance was positively associated with richness, contrary to the patterns found for all hemiparasites and the perennial‐only *Pedicularis* (Appendix S1: Figures S5 and S6). These patterns may hint at potential differences between annual and perennial hemiparasites. This issue underscores the importance of identifying taxa to species whenever possible during vegetation surveys. This practice would enhance the potential for interdisciplinary studies.

## CONCLUSION AND BROADER IMPLICATIONS

This study improves our understanding of the role that hemiparasitic plants, as a functional group, play in structuring their communities. This is the largest‐scale analysis of its type, spanning multiple ecosystems and hemiparasitic genera, and the first on North American flora. The illustrated trends between hemiparasites and evenness would be strengthened by additional manipulative research into both the physiology and ecology of these genera.

Research investigating any differences in hemiparasite physiology as influenced by life history could clarify and reveal any species‐specific impacts of hemiparasites on their communities. Furthermore, we should prioritize determining the key traits of hemiparasitic species that impact communities, as well as the key traits of suitable hosts. This is particularly needed for the understudied genera examined here, for which we have little anatomical and physiological research, especially compared to hemiparasitic genera like *Rhinanthus*. Determining these functional traits would provide valuable insight into the nuances on the functional role of hemiparasites and clarify their potential utility in ecological restoration.

## AUTHOR CONTRIBUTIONS

Jasna Hodžić conceived of the idea. Jasna Hodžić, Ian Pearse, and Jonathan D. Bakker conducted analyses. Jeffrey D. Corbin and Evelyn M. Beaury compiled and cleaned the data. Jasna Hodžić wrote the first draft of the manuscript, and all authors contributed substantially to revisions.

## CONFLICT OF INTEREST

The authors have no conflicts of interest to declare.

## Supporting information


Appendix S1

## Data Availability

Data and code (jhodzic17, 2022) are available in Zenodo at https://doi.org/10.5281/zenodo.6618403.
