# Supplementary material for: Root hemiparasitic plants are associated with more even communities across North America
Source: Ecology. 2022 Sep 30;103(12):e3837. doi: 10.1002/ecy.3837 (PMC10077900; doi:10.1002/ecy.3837)
Supplement: Supplementary file 1 — Appendix S1 [file ECY-103-0-s001.pdf]

Appendix S1

Root hemiparasitic plants are associated with more even communities across North America

Jasna Hodžić, Ian Pearse, Evelyn M. Beaury, Jeffrey D. Corbin, Jonathan D. Bakker

Journal: Ecology

Table S1: Descriptive statistics of all plant growth forms (herbaceous plants, woody plants, and all plants) and hemiparasites for plots used in paired plot presence analysis (n = 1,608). Abundance was calculated as the sum of the percent cover. Plots are sorted based on whether hemiparasites are present or absent. Percent of plots present refers to what percent of total plots contains the given growth-form.

	Hemiparasite Presence							
	Present				*Absent*			
	Percent of Plots present	Mean Abundance	Minimum Richness	Maximum Richness	Percent of Plots present	Mean Abundance	Minimum Richness	Maximum Richness
Hemiparasites	100	1.28	1	3	-	-	-	-
Herbaceous Plants	100	53.4	1	72	99	46.6	0	56
Woody Plants	92	40.2	0	37	91	62.2	0	31
All Plants	100	93.6	5	111	100	108.8	2	74

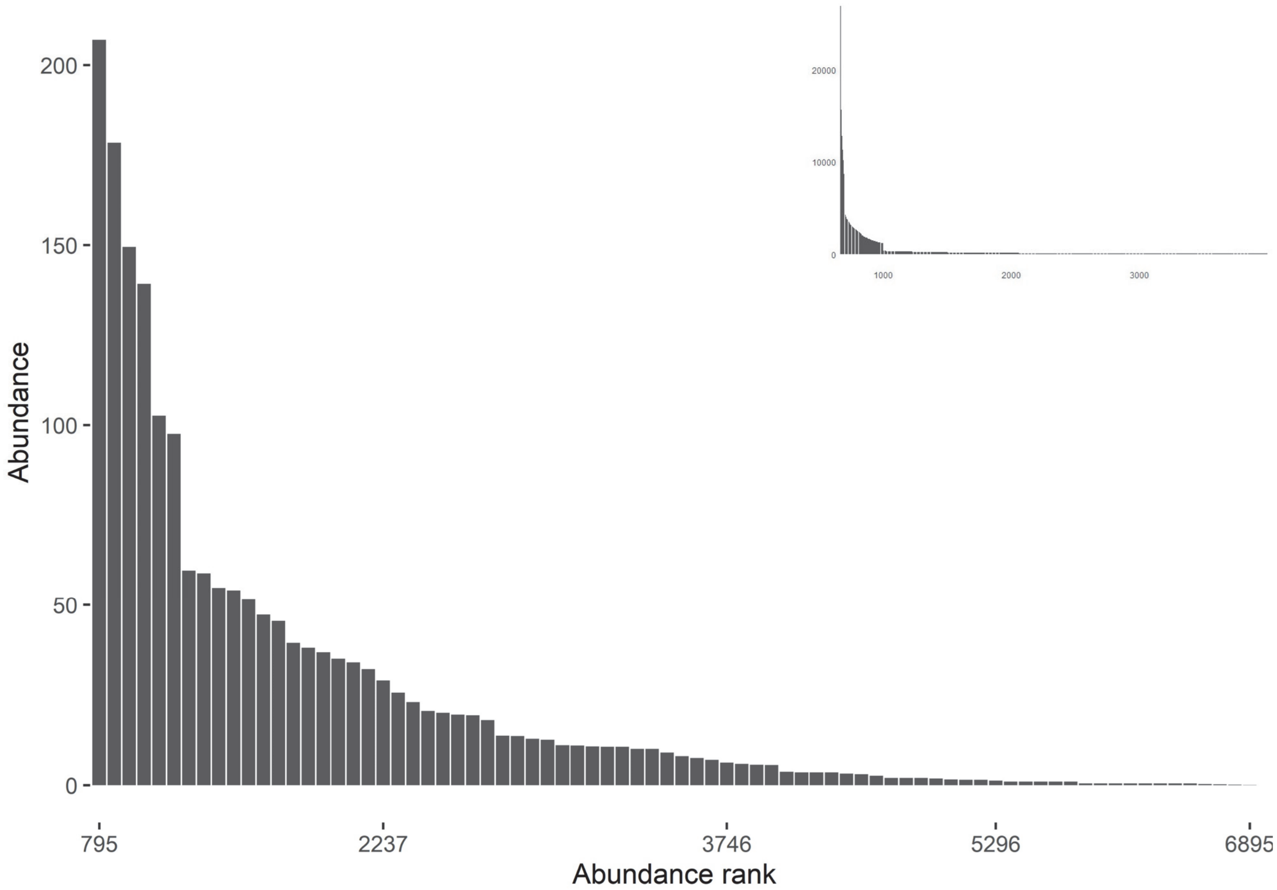

Figure S1: Rank abundance curve of 78 hemiparasitic plant taxa in our dataset showing the abundance and abundance rank, with the most abundant species given the lowest rank. Hemiparasite taxa were between ranks 795 and 6,895. The inset shows the rank abundance curve for the entire dataset of 6,9872 plant species.

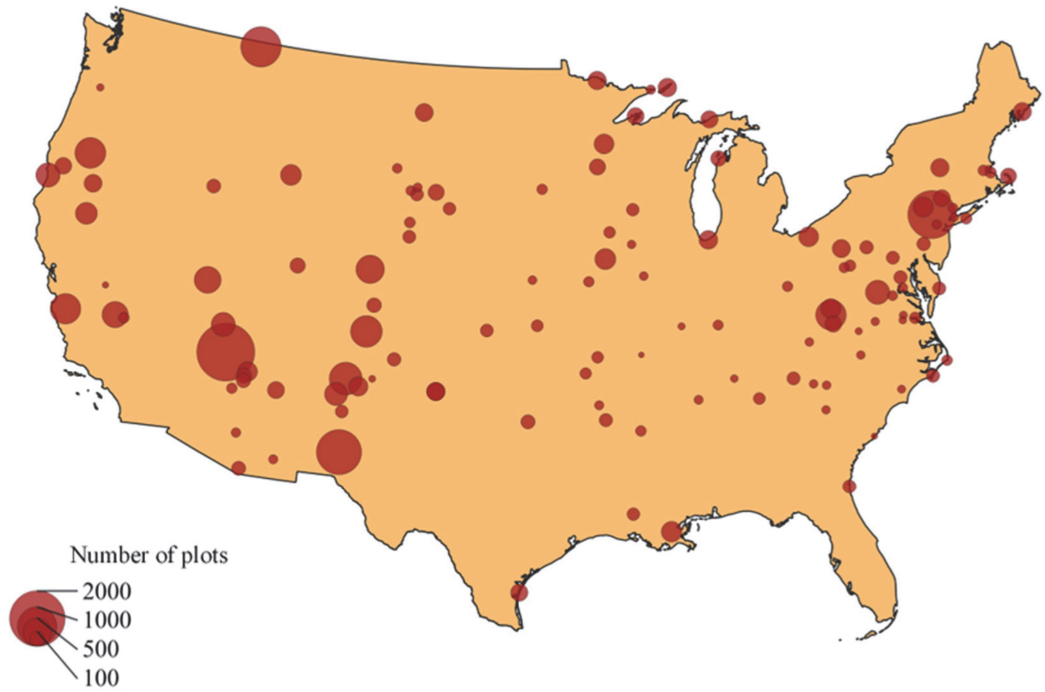

Figure S2. Map of the conterminous United States. Red circles indicate the location of 129 management units, scaled by the number of plots per management unit.

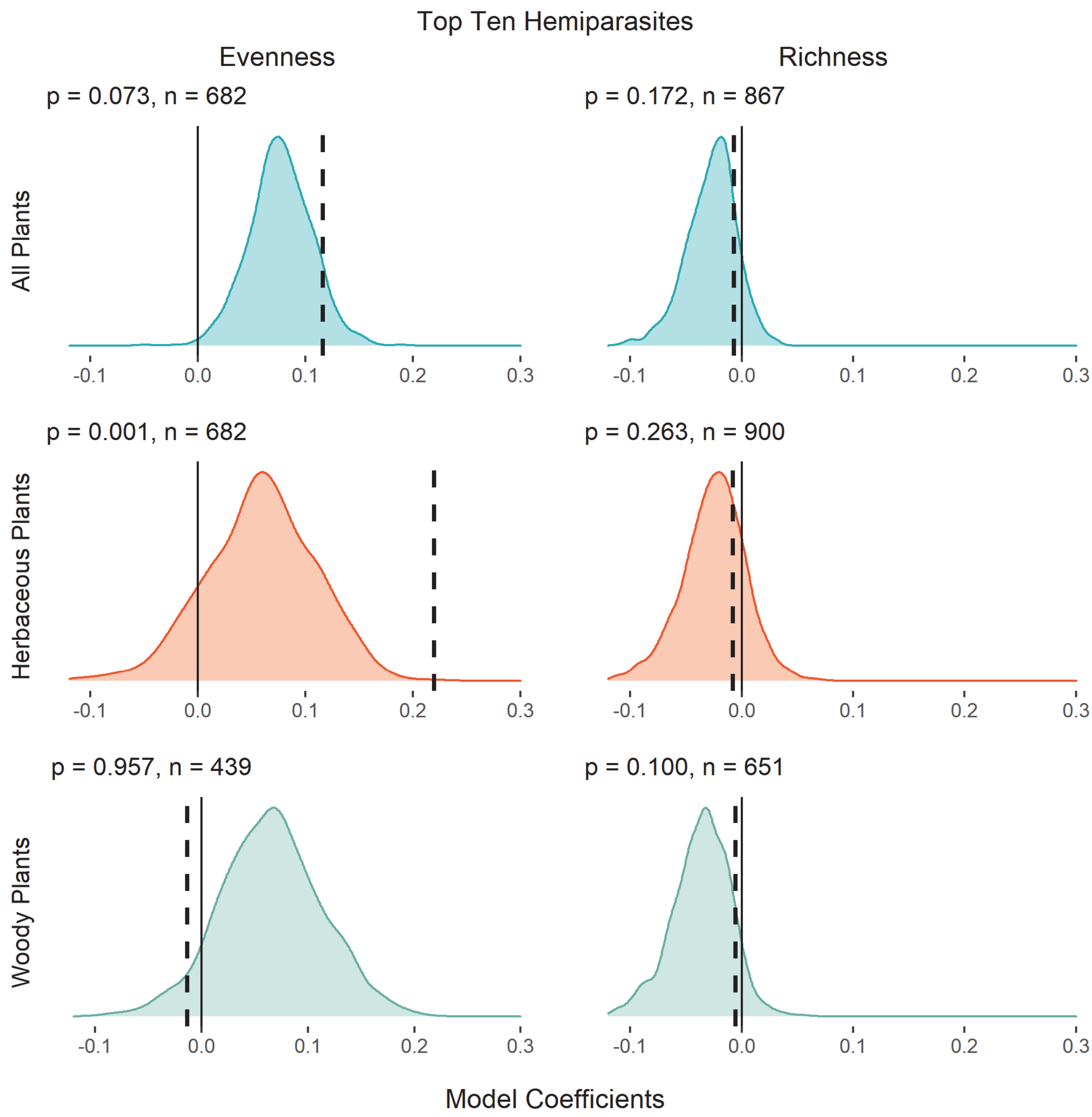

Figure S3*:* Density graph of model coefficients for 1,000 abundance null models created from plants (n = 887) in the same rank abundance (795–1,691) as the top ten most abundaunt hemiparasites (genera: *Castillea, Pedicularis, Krameria* and *Comandra*) in the dataset for community evenness (left) and richness (right) for all plants (top row), herbaceous plants (midde row), and woody plants (bottom row). We excluded plots in which the focal hemiparasite taxa were present with other hemiparasite species. We dropped all models that had singular solutions

from the analysis; the sample size for each analysis is indicated next to the p-value. The y-axis limits are identical across rows. The dashed line indicates the model coefficient for hemiparasites; positive coefficients indicate a positive relationship between abundance and the response; negative coefficients indicate a negative relationship. Statistical significance was calculated as the proportion of null models with a coefficient equal to or greater than that for hemiparasites, and is reported above each graph. The solid line at zero indicates no relationship between focal plant abundance and the response.

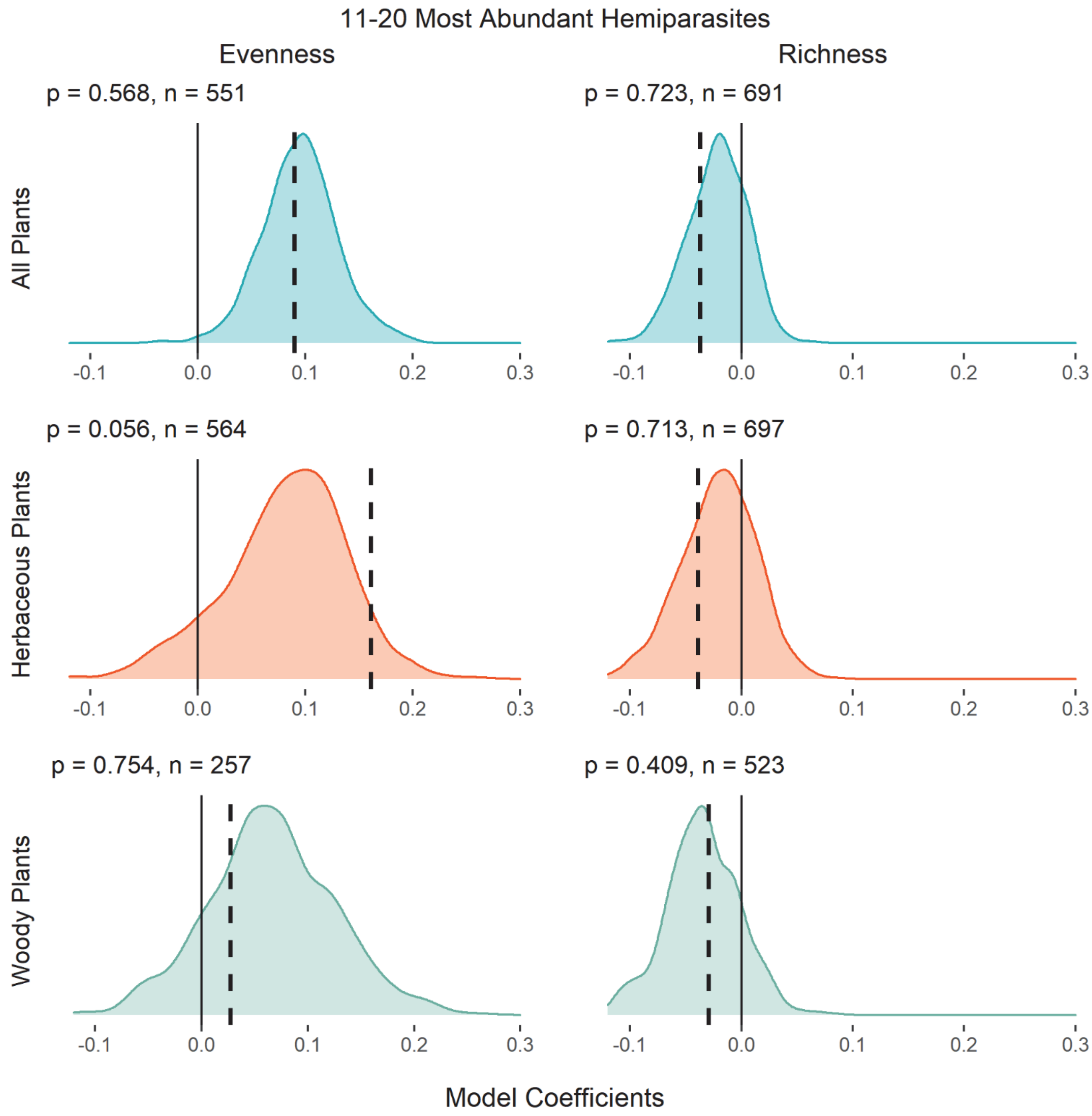

Figure S4: Density graph of model coefficients for 1,000 abundance null models created from plants (n = 413) in the same rank abundance (1,729–2,141) as the top ten most abundaunt hemiparasites (genera: *Castillea, Pedicularis, Cordylanthus* and *Geocaulon*) in the dataset for community evenness (left) and richness (right) for all plants (top row), herbaceous plants (midde

row), and woody plants (bottom row). We excluded plots in which the focal hemiparasite taxa were present with other hemiparasite species We dropped all models that had singular solutions from analysis; the sample size for each analysis is indicated next to the p-value. The y-axis limits are identical across rows. The dashed line indicates the model coefficient for hemiparasites; positive coefficients indicate a positive relationship between abundance and the response; negative coefficients indicate a negative relationship. Statistical significance was calculated as the proportion of null models with a coefficient equal to or greater than that for hemiparasites, and is reported above each graph. The solid line at zero indicates no relationship between focal plant abundance and the response.

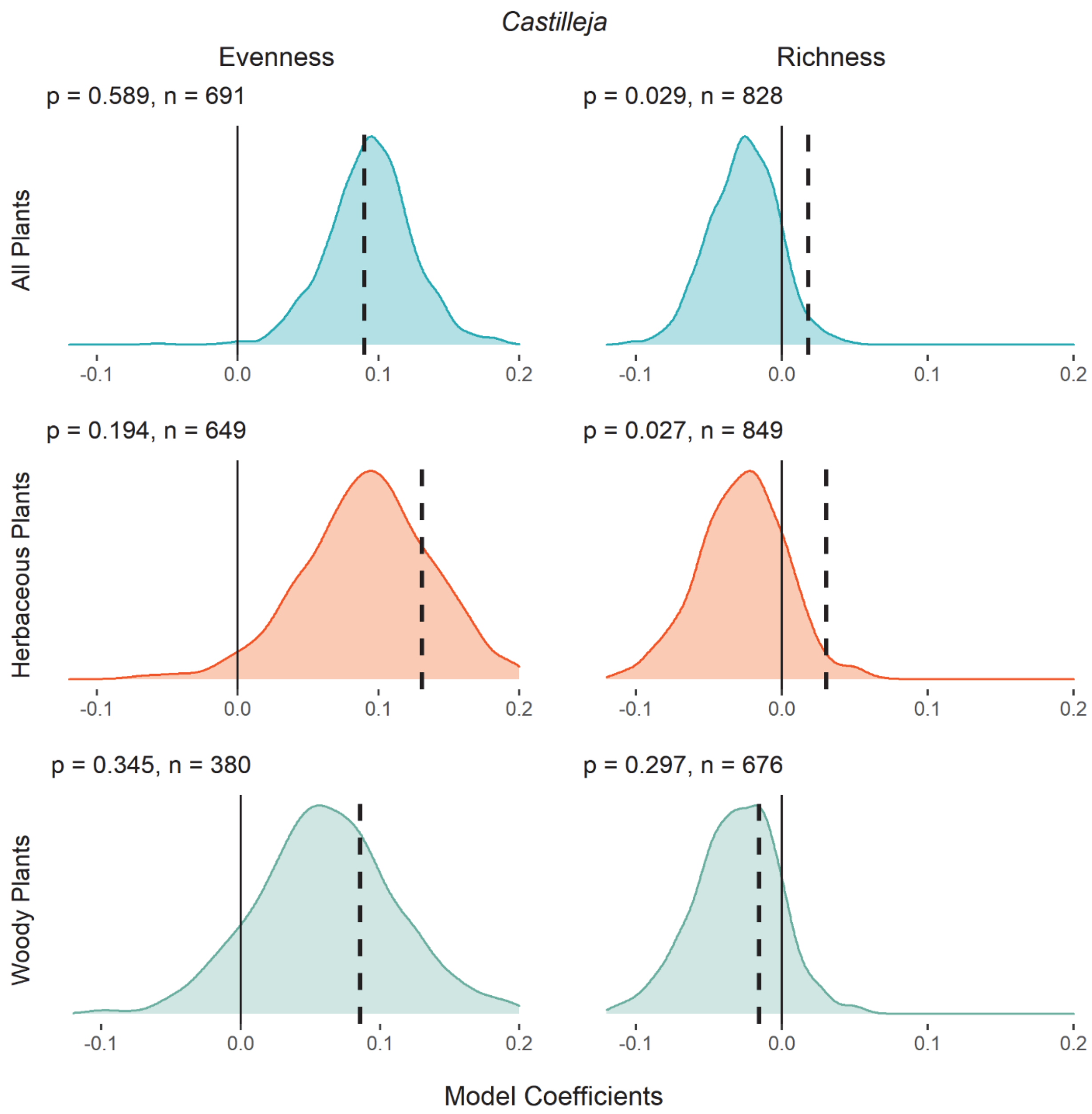

Figure S5: Density graph of model coefficients for 1,000 abundance null models created from plants (n = 5,628) in the same rank abundance (1,256–6,547) as *Castilleja* hemiparasites (n = 34 taxa) for community evenness (left) and richness (right) for all plants (top row), herbaceous plants (midde row), and woody plants (bottom row). We excluded plots in which *Castilleja* was present with another hemiparasite species. We dropped all models that had singular solutions from analysis; the sample size for each analysis is indicated next to the p-value. The y-axis limits

are identical across rows. The dashed line indicates the model coefficient for hemiparasites; positive coefficients indicate a positive relationship between abundance and the response; negative coefficients indicate a negative relationship. Statistical significance was calculated as the proportion of null models with a coefficient equal to or greater than that for hemiparasites, and is reported above each graph. The solid line at zero indicates no relationship between focal plant abundance and the response.

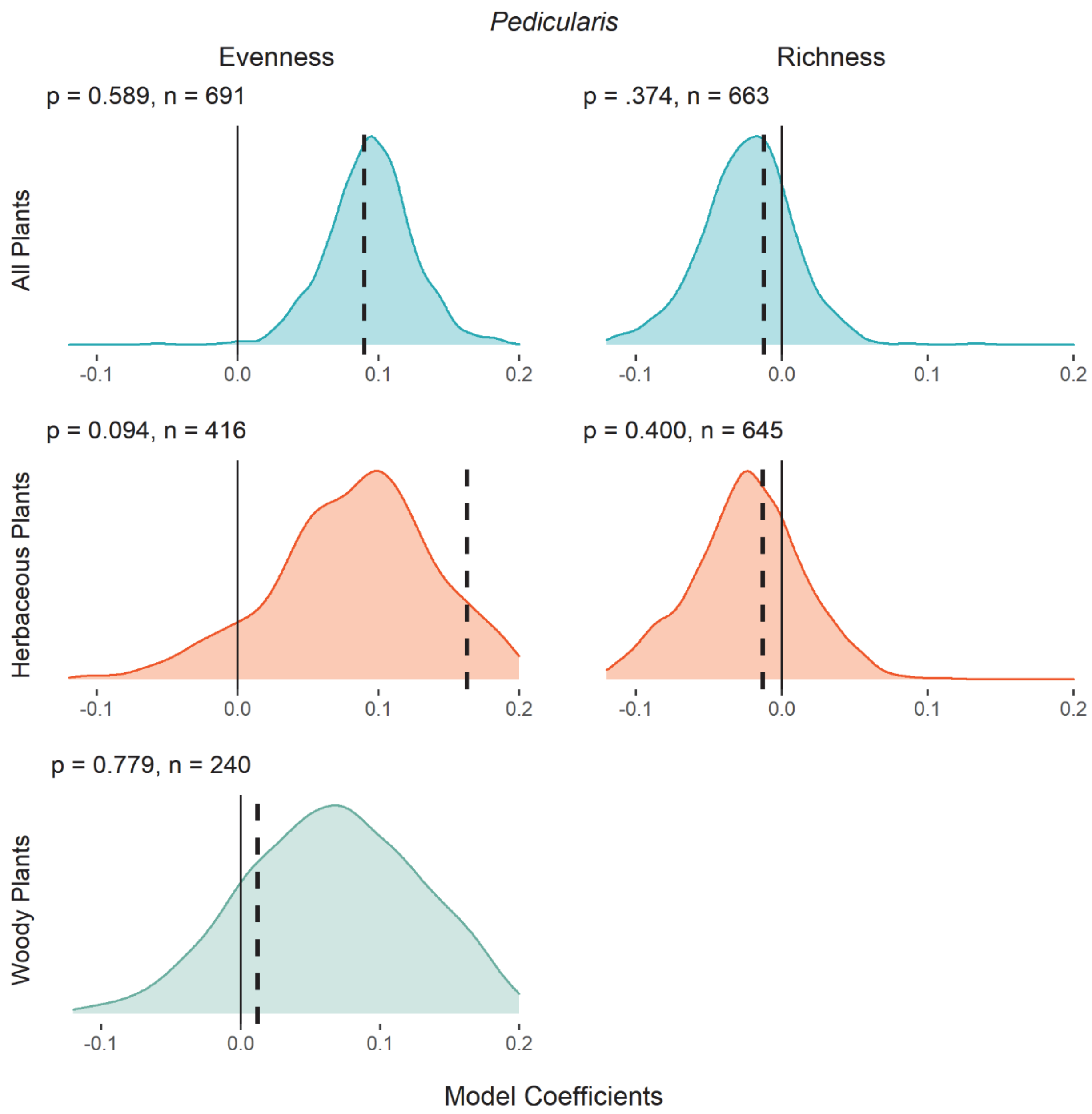

Figure S6: Density graph of model coefficients for 1,000 abundance null models created from plants (n =5,790 taxa) in the same rank abundance (970–6,776) as *Pedicularis* hemiparasites (n = 17 taxa) for community evenness (left) and richness (right) for all plants (top row), herbaceous plants (midde row), and woody plants (bottom row). We excluded plots in which *Castilleja* was present with another hemiparasite species. We dropped all models that had singular solutions from analysis; the sample size for each analysis is indicated next to the p-value. All models for

woody plant richness had singularity issues and we dropped this variable from analysis. The y-axis limits are identical across rows. The dashed line indicates the model coefficient for hemiparasites; positive coefficients indicate a positive relationship between abundance and the response; negative coefficients indicate a negative relationship. Statistical significance was calculated as the proportion of null models with a coefficient equal to or greater than that for hemiparasites, and is reported above each graph. The solid line at zero indicates no relationship between focal plant abundance and the response.
